# DNA Damage Response Pathways in Dinoflagellates

**DOI:** 10.3390/microorganisms7070191

**Published:** 2019-07-05

**Authors:** Chongping Li, Joseph Tin Yum Wong

**Affiliations:** 1 Department of Ocean Science, The Hong Kong University of Science and Technology, Clearwater Bay, Kowloon, Hong Kong, China; 2 Division of Life Science, The Hong Kong University of Science and Technology, Clearwater Bay, Kowloon, Hong Kong, China

**Keywords:** dinoflagellate, DNA damage, DNA repair

## Abstract

Dinoflagellates are a general group of phytoplankton, ubiquitous in aquatic environments. Most dinoflagellates are non-obligate autotrophs, subjected to potential physical and chemical DNA-damaging agents, including UV irradiation, in the euphotic zone. Delay of cell cycles by irradiation, as part of DNA damage responses (DDRs), could potentially lead to growth inhibition, contributing to major errors in the estimation of primary productivity and interpretations of photo-inhibition. Their liquid crystalline chromosomes (LCCs) have large amount of abnormal bases, restricted placement of coding sequences at the chromosomes periphery, and tandem repeat-encoded genes. These chromosome characteristics, their large genome sizes, as well as the lack of architectural nucleosomes, likely contribute to possible differential responses to DNA damage agents. In this study, we sought potential dinoflagellate orthologues of eukaryotic DNA damage repair pathways, and the linking pathway with cell-cycle control in three dinoflagellate species. It appeared that major orthologues in photoreactivation, base excision repair, nucleotide excision repair, mismatch repair, double-strand break repair and homologous recombination repair are well represented in dinoflagellate genomes. Future studies should address possible differential DNA damage responses of dinoflagellates over other planktonic groups, especially in relation to possible shift of life-cycle transitions in responses to UV irradiation. This may have a potential role in the persistence of dinoflagellate red tides with the advent of climatic change.

## 1. Introduction

Cellular DNA is continuously challenged with intracellular or exogenous agents that cause DNA damages. To preserve DNA integrity during cell division, cells have developed integrated signaling cascades linking DNA damage responses (DDRs) to cell-cycle transition, making judgement call as to damage repair per proliferation versus growth postponement, life-cycle transitions or cell death. DNA repair systems are generally conserved in nucleosomal eukaryotes.

Dinoflagellates are a major phytoplankton group in aquatic ecosystems, contributing significantly to ocean primary productivity and carbon cycling [1]. Members of the group are infamous for causing harmful algal blooms (red tides) [2], which may cause mortality or physiological impairment either due to toxin production, oxygen depletion or physical clotting of gills attributed to high biomass [3]. Proliferation of many dinoflagellates is favored with prolonged surface water stratification with the increase in seawater temperature, and global warming was predicted to expand the distribution of harmful algal blooms into higher latitudes [4]. An active cell death called paraptosis, different from apoptosis, was also observed in *Amphidinium carterae* under culture senescence and darkness [5], which implicated strong solar energy could differentially modulate dinoflagellate production relative to other eukaryotic groups. In addition to their ecological profoundness, dinoflagellates behold the only alternative chromosomal packaging system in their Liquid Crystalline Chromosomes (LCCs), composing of superhelical modules and cation-mediated anisotropic organization (reviewed in this issue [6]). LCCs are characterized by many unique features including no detectable architectural nucleosomes and substantial replacement of thymine with 5-hydroxymethyluracil (reviewed in this issue [6]), implicating they may have different susceptibility to DNA damage agents when compared with the typical nucleosomal chromosome structure. Likely attributed to susceptibility of LCCs’ anisotropic organization and their large chromosomes (many up to microns, and genome among the largest in eukaryotes), many dinoflagellates are susceptible to physical turbulences of the sea, with many species arresting in cell cycle in response to mechanical stresses [7]. The rising ocean temperature has been associated with loss of symbiotic dinoflagellates in corals and other invertebrates, which are co-exposed to increasing physical stresses in the subtidal-littoral zones, with increasing climatic extremes [8,9].

UV irradiation inhibited growth and motility of dinoflagellates *Gyrodinium aureolum*, *Prorocentrum minimum* and *Heterocapsa triquetra* [10,11], *Symbiodinium californium* and *Symbiodinium microadriaticum* [12]. UV irradiation caused chromosome breaks in *Prorocentrum micans* [13], despite not being characterized at the molecular level in dinoflagellates. There is a paucity of data as to the effect of DNA damage on cell-cycle delay, amid inhibition of proliferation. With this imperative, we conducted a global analysis of the DNA damage repair genes in dinoflagellates, from different transcriptomic databases, and compared to other eukaryotic orthologues. Most dinoflagellate species are capable of mixotrophy [14,15], implicating photo-inhibition (or sub-eutrophic zone) as a non-limiting factor to the group, which can be a common factor that modulates selective cell proliferation of surface groups, especially if strong irradiation were to cause cell deaths in other groups. Differential effects on different phytoplankton groups have been a recognized driver for future bio-oceanographic regimes in response to climatic changes [16,17]. The unique architectural organization of dinoflagellate liquid crystalline chromosomes will substantiate differences in their DDRs to increasing UV irradiation, acidity, and physical turbulences.

## 2. Materials and Methods

Orthologues (ORFs) of DDR proteins (the complete lists of these proteins are available from: https://www.mdanderson.org/documents/Labs/Wood-Laboratory/human-dna-repair-genes.html#Human%20D) from animal *Homo sapiens* and budding yeast *Saccharomyces cerevisiae* were retrieved from UniProt database and used as reference sequences. These protein sequences were queried against the transcriptome datasets of *Crypthecodinium cohnii* (unpublished datasets, the final extracted sequences are available in Table S1), *Symbiodinum minutum* [18] (published data from: https://marinegenomics.oist.jp/symb/viewer/info?project_id=21), and *Lingulodinium polyedrum* ([19], the final obtained sequences are available in Table S1) by tBLASTn algorithm with a cut-off E-value of 1e-5 using the TBtool software [20]. The three dinoflagellate species represent heterotrophic, symbiotic and autotrophic dinoflagellate species respectively. For reference polypeptides with no hits, we further included orthologues of fission yeast *Schizosaccharomyces pombe* and plant *Arabidopsis thaliana* as reference templates to query against the transcriptome datasets. The hit sequences were extracted out and further verified by running a BLASTX algorithm against NCBI non-redundant (nr) database. If the reference genes or its orthologues appeared on the top five reciprocal BLAST hits, we label herewith as an orthologue. Given the special features of LCCs and the vast evolutionary distances, further studies would be required to functionally characterize these orthologues.

Phylogenetic analysis based on neighbour-joining and maximum-likelihood algorithms were conducted using software MEGA 5.05 [21], and only nodes with bootstrap value over 0.5 (50%) were labelled. Cladding of phylogenetic groups with major expected sister groups, though not a proof, gives additional information on expected evolutionary rates; long branches, on the other hand, would be indicative of accelerated evolution.

## 3. Results and Discussion

### 3.1. DNA Damage Checkpoint Signaling Networks

DNA damage checkpoint signaling is initiated by two conserved apical regulators Ataxia telangiectasia mutated (ATM) and ataxia telangiectasia mutated and Rad3-related (ATR), which are members of the phosphoinositide 3-kinase-related protein kinase (PIKKS) family, acting as major switches in DNA damage repair or apoptosis, senescence and even cell death [22,23,24,25].

In mammalian cells, ATM mainly responds to double-stranded DNA breaks (DSBs) generated by ionizing radiation [26]. The Mre11-Rad50-Nbs1 (MRN) complex recognizes the DSBs and stimulates the activation of ATM, which then triggers the rapid phosphorylation of the C-terminal tail (Ser 139) of the histone variant H2AX [27,28]. The phosphorylated histone variant γH2AX then interacts with Mdc1 through its C-terminal BRCT domain, which recruits more MRN complex and ATM, reinforcing γH2AX phosphorylation, which is taking as a common hallmark of DNA damage [29,30].

The ATR pathway is primarily triggered by replication protein A (RPA) coated single-stranded DNAs (ssDNAs) resulted from replication stress or UV exposure and other genotoxic agents [22], which recruits ATR-interacting protein (ATRIP) and ATR together to the lesion sites. The activation of ATR is mediated by ATR activators. TopBP1 is one of these ATR activators, which is also conserved in different organisms [31]. Its recruitment depends on the PCNA-like Rad9-Rad1-Hus1 (9-1-1) checkpoint clamp complex [32,33].

**Figure 1:**
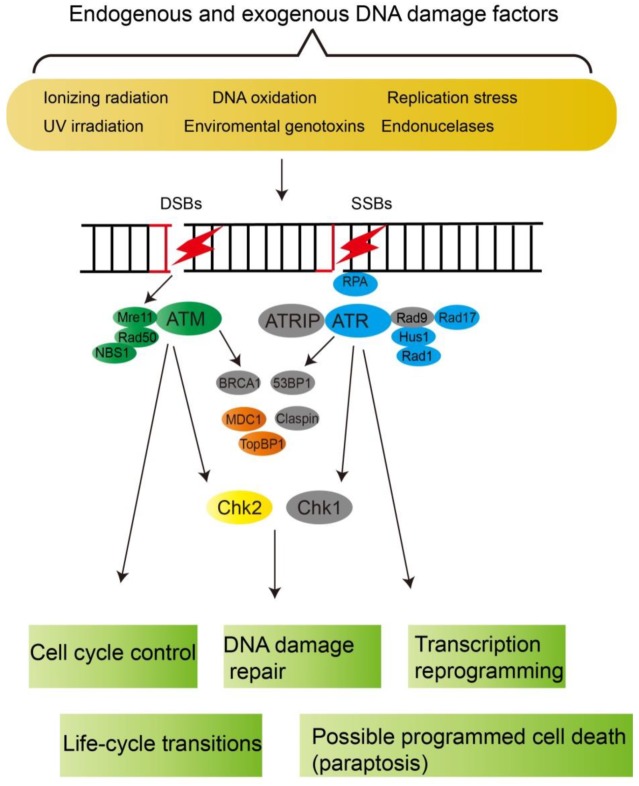
Diagrammatic summary of the DNA damage response signaling network. The grey ellipses denote absence of putative dinoflagellate orthologues, whereas other colors indicate presence of putative dinoflagellate orthologues. For simplicity, nomenclatures differentiating genes, proteins and mutations are not enforced in this study.

Following activation, ATM and ATR phosphorylates downstream proteins to amplify the signaling cascade for coordination of cell cycle, DNA repair and replication. A key amplification point is the two effector kinases, Chk2 and Chk1, two ATM/ATR substrates, which are cell-cycle control proteins: including phosphorylation of the cell-cycle phosphatase Cdc25, leading to cyclin-dependent kinase (CDK) inactivation and halting cell cycle [34,35,36,37]. Chk1 and Chk2 are conserved in metazoan and fungi, but both Chk1 and Chk2 orthologues are not present in plant kingdoms [38]. Chk1 and Chk2 have many overlapped substrates and non-overlapping substrates in different eukaryotes [39]. Although a previous study reported that Chk1 was found in *Symibodinum* and *Lingulodinium* [40], our reciprocal BLAST analysis showed that these putative genes were not true Chk1 orthologues. It seems that only Chk2 is present in dinoflagellates (Figure 1 and Table 1).

Further down the signaling cascade (Figure 1 and Table 1), orthologues of some ATM accessory proteins MDC1, 53BP1, but not BRCA1, were found in dinoflagellate transcriptomes [26,41]. BRCA1 is only present in animals and plants [42]. Therefore, it is not unexpected to have no BRCA1 in dinoflagellates. Both orthologues of TopBP1 and Claspin, accessory proteins for ATR [24,25], are absent from our bioinformatics analysis.

Except for the ATRIP and Rad9, all other upstream factors including the central kinase ATM and ATR were found in *C. cohnii*, *S. minutum* and *L. polyedrum* (Figure 1 and Table 1). ATRIP, an obligate partner of ATR, and Rad9-Hus1-Rad1 complex, play an essential role for the recognition of RPA-ssDNA and subsequent activation of the ATR signaling respectively [24]. Therefore, the absence of ATRIP and Rad9 is surprising, which is probably due to sequence divergence. Phylogenetic analysis of the ATM and ATR of dinoflagellates suggested they formed a single clade respectively and clustered together with the apicomplexa (Figure S1A,B), consistent with their phylogenetic relationship under the super phylum alveolate [43]. Further investigations should address the bridging pathways between switches between vegetative growth, cell-cycle arrest and life-cycle transitions. These pathways would likely have group-specific genes specially adapted to dinoflagellate ecological niches.

### 3.2. DNA Repair Pathways

#### 3.2.1. Direct Reversal of DNA Lesion

**Figure 2:**
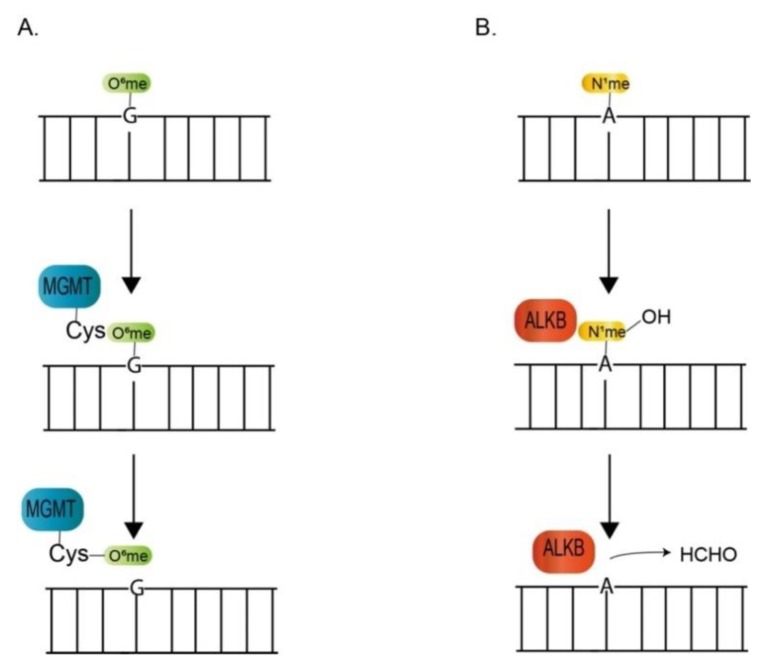
Diagrammatic summary of dinoflagellate orthologues in the direct reversal of DNA damage pathway. (**A**) Repair of O^6^-methylguanine by the MGMT protein. (**B**) Repair of N^1^-methyladenine and N^3^-methylcytosine by ALKB protein. Only N^1^-methyladenine is represented.

Alkylating agents—widely distributed reactive chemicals in intracellular and extracellular environments—react with DNA and produce various kinds of modifications on the DNA bases and backbone, leading to structure alterations and functional disruptions [44,45,46]. The alkylation attack on DNA mainly occurs at the ring nitrogen (N) and extracyclic oxygen (O) atoms of the DNA bases [46,47]. O^6^-methylguanine (O^6^-meG), a major deleterious base adduct produced from the reaction with the O^6^ position of guanine, will elicit a mispair with thymine during DNA duplication, causing the transition mutation of G:C to A:T. The O^6^-alkylguanine-DNA alkyltransferase, the Ada protein in *E. coli* and MGMT/AGT protein in mammalians, is responsible for direct repair of this type of lesion (Figure 2A). During repair process, alkyl group of O^6^-meG is transferred to Cys residues of MGMT protein, leading to MGMT protein’s inactivation and degradation [48]. N^1^-methyladenine (N^1^-meA) and N^3^-methylcytosine (N^3^-meC) are two other kinds of lesions occurred in the exposed DNA base of single-stranded DNA or replication fork. ALKB proteins, members of α-ketoglutarate/iron (II)-dependent dioxygenases, are involved in reversing these types of DNA lesions through oxidative dealkylation of the alkyl groups from N^1^-meA and N^3^-meC, leading to hydroxylmethylated products and the subsequent release of formaldehyde and the repaired base (Figure 2B) [47,49,50]. The orthologues of MGMT and ALKB protein are found in dinoflagellates transcriptomes (Table 2). The modified base *N*6-methyladenine (6mA), the most abundant modified base in eukaryotic RNAs [51,52], is also present naturally in DNA of dinoflagellates chromosomes, which was reported to accounting for 2–3% of the total nucleotides [53,54]. 6mA, a major type of modification in bacteria, is associated with restriction-modification system and discrimination between original and newly produced DNA [55]. 6mA is also conserved in eukaryotic genomes and linked with gene regulation events [56,57,58,59,60]. Both bacterial and human AlkB protein have activities for demethylating 6mA [60,61]. It remains to be further determined if dinoflagellate AlkB protein has activities towards 6mA.

#### 3.2.2. Photoreactivation

Photoreactivation, regarded as the most efficient and error-free pathway for reversal of UV-induced DNA damage, is present in bacterial, fungi, animals (except the placental mammals) and plants [62]. In plants, photoreactivation is the major and preferred pathway responsible for UV-induced lesions repair [63,64]. Photoreactivation depends on a single light-activated enzyme called photolyase to recognize and repair the photoproducts cyclobutane-pyrimidine dimers (CPDs) or 6-4 pyrimidine–pyrimidine photoproducts (6-4PPs) at lesion sites [65]. CPD photolyases and (6-4) photolyases, specific for the repair of CPDs and 6-4PPs respectively, belong to the cytochrome/photolyase family (CPF) and share similar biochemical activities on their substrates [66]. Those photolyases directly bind to damaged DNA substrates and rely on light-dependent reduction of their cofactor flavin adenine dinucleotide (FAD) to mediate lesion repair [67,68]. The activated FADH^-^ then passes an electron to the lesions for the cleavage of the covalent bonds within pyrimidine dimers. In addition, Cry-DASH proteins, another group in CPF, are also suggested to be a group of single-stranded DNA photolyases [69,70,71]. They could bind to damaged sites of ssDNAs and have a preference for the repair of CPDs. Our bioinformatic analysis indicated putative orthologues are present in dinoflagellates (Table 2). Dimerization of 5-hydroxymethyluracil (5hmu), the major UV-induced adduct, is theoretically impossible; as 5hmu replaces substantial amount, but not all, genomic thymines, photoreactivation repair in LCCs is likely compartmentalized.

#### 3.2.3. Three Excision Repair Pathways, Nucleotide Excision Repair (NER), Base Excision Repair (BER), and DNA Mismatch Repair (MMR), Confer Single-Strand DNA Damage Repair Through Excision-Coupled Resynthesis

BER and MMR pathways deal with non-bulky lesions in DNA, which are glycosylase-dependent and mismatch base-pair dependent respectively. NER pathway copes with the bulky DNA lesions, which is TFIIH protein complex-dependent. Althouth detailed mechanisms are different among these three excision repair pathways, they share some similar components of DNA resynthesis, including PCNA and DNA polymerase δ.

##### Base Excision Repair (BER)

**Figure 3:**
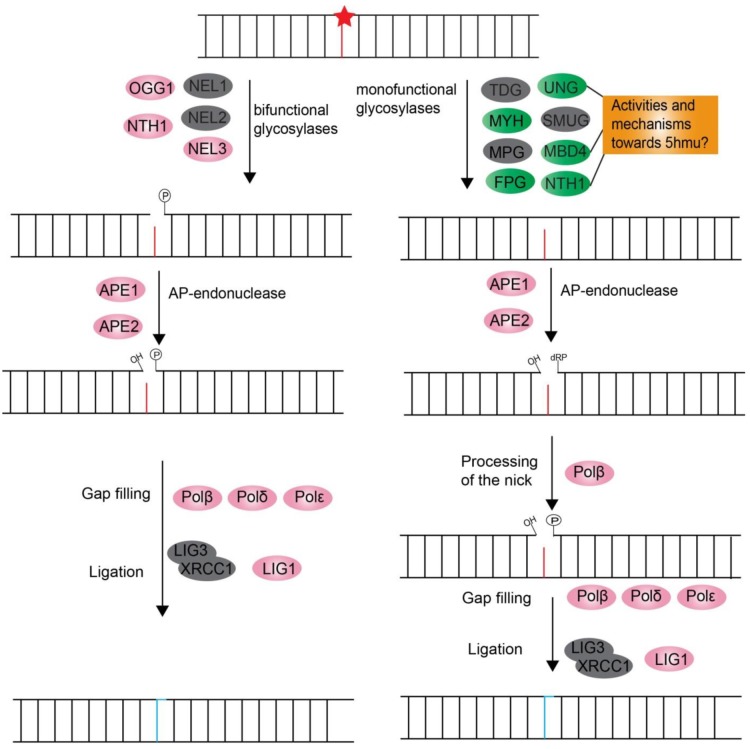
Diagrammatic summary of dinoflagellate orthologues predicted in the base excision repair pathway. The ellipses filled with grey color mean the absence of putative dinoflagellate orthologues in the searched transcriptomes. The other colors in ellipses indicate the presence of putative dinoflagellate orthologues.

BER rectifies a wide range of DNA damages that modify non-bulky bases such as DNA oxidation from reactive oxygen species (ROS) attack, hydrolysis, deamination and alkylation [72,73,74,75]. Impaired DNA bases are identified and removed by DNA glycosylases, generating an abasic (apurinic-apyrimidinic, AP) site in DNA. The dinoflagellates have both mono-functional and bi-functional glycosylases (Figure 3 and Table 3). The glycosylases targeting uracil and its derivatives including UNG [76], SMUG1 [77], MBD4 [78], TDG [77,78] and NTH1 [79], are of special interest. These genes were reported to have activities against 5hmu. The modified base 5hmu is a natural component of the DNA of dinoflagellates, which could replace 12–70% of thymine in genomes of dinoflagellates [53,54,80]. The existence of UNG, MBD4 and NTH1 implicates dinoflagellates must develop a mechanism to distinguish between the damage-induced and endogenous 5hmu, or a specific compartmentalization mechanism. The AP-site generated by mono-functional DNA glycosylases is targeted by AP-endonuclease (APE1), which then produces a single nucleotide nick, leading to the 3′OH and 5′deoxyribosephosphate(dRP) terminal in the DNA backbone. DNA polymerase Polβ is then engaged to remove the 5′-dRP group and produce 3′OH and 5′P ends. Alternatively, the bi-functional DNA glycosylases could cut the phosphodiester bond of the AP-site directly through its AP lyase activity and also create a single nucleotide nick. The nick is further converted into 3′OH and 5′P ends by extra enzymatic activities such as APE1 or polynucleotide kinase (PNKP). Later, the DNA polymerase Polβ and ligase LIG3/XRCC1 complex or ligase LIG1 is involved in gap-filling DNA synthesis and ligation sequentially. Additionally, the long-patch BER pathway is used to cope with 2–12 nucleotides lesions, in which DNA polymerases δ and ε (Polδ, Polε), FEN1, PCNA and DNA ligase I are involved [81,82]. For these steps, LIG3 and XRCC1 were not found in dinoflagellates (Figure 3 and Table 3). No genes equivalent to LIG3 were found in many eukaryotes including most plants and budding yeast [83]. Pol λ and LIG1 were functionally replaced by Polβ and LIG3 in plant *Arabidopsis thaliana* [84]. A similar mechanism could be adopted by dinoflagellates.

##### Nucleotide Excision Repair

Nucleotide Excision Repair (NER) is a multi-step process that recognizes and removes bulky structurally unrelated DNA lesions, such as CPDs and 6–4PPs induced by UV irradiation, endogenous oxidative DNA damage, and DNA adducts formed by environmental and chemical mutagens [85,86,87,88,89,90]. NER consists of two pathways: the global genome NER (GG-NER) and the transcription-coupled NER (TC-NER). GG-NER occurs in all regions of genomic DNA, while TC-NER is dedicated to repairing lesions that block the transcription of active genes. The two pathways adopt different strategies to initiate DNA damage recognition but share the same sets of proteins in the later stages of the repair process. In mammalian cells, with the help of UV-damaged DNA-binding complex composed of DDB1 and DDB2 subunits, XPC-Rad23B complex (with CETN2) is responsible for the recognition of the DNA lesions in GG-NER pathway [87,91]. Stalled RNA polymerase activates the TC-NER pathway and recruits CSA and CSB proteins to remove them at lesion sites [92,93]. TFIIH protein complex is involved in the next steps of the GG-NER and TC-NER pathways for DNA lesion verification, helix unwinding and incision. TFIIH comprises of 10 subunits and can be divided into a core complex consisting of XPB, XPD, p8, p34, p44, p52 and p62, and a cyclin-activated kinase (CAK) complex containing MAT1, CDK7 and cyclin H [94,95,96]. The helicase and ATPase activities of XPB and XPD are required for unwinding the DNA into a bubble-like structure at the lesions. Later, the pre-incision complex consisting of XPA, RPA, and XPG is formed at the damaged DNA sites, which help to recruit endonucleases. Incision action is thought to be initiated by the 5′ incision complex ERCC1-XPF, followed by 3′ incision of XPG, creating a single-strand DNA break. DNA polymerase δ, ε, and κ (Polδ, Polε and Pol κ), with the assistance of PCNA and RFC, are required for the subsequent DNA repair synthesis. DNA ligase LIG1 or LIG3/XRCC1 complex are involved in the final gap filling after DNA repair synthesis.

**Figure 4:**
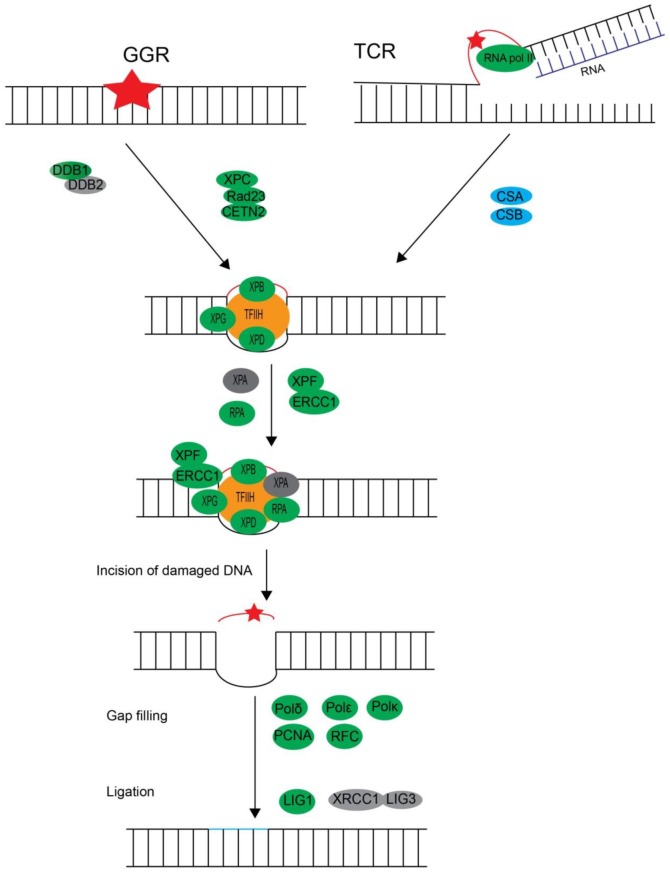
Diagrammatic summary of the dinoflagellate orthologues predicted in the nucleotide excision repair pathway. The ellipses filled with grey color mean the absence of putative dinoflagellate orthologues in the searched transcriptomes. The other colors in ellipses indicate the presence of putative dinoflagellate orthologues.

Except DDB2, XPA, the subunits of TFIIH complex including GTF2H1 and GTF2H5, and the RPA3 subunits of RPA complex, the other proteins involved in NER pathway were identified in dinoflagellates (Figure 4 and Table 4). XPA, an intrinsically unstructured protein, is a crucial component of the pre-incision complex for DNA damage recognition [97]. No putative orthologues were found in plants, although they have the ability to remove UV photoproducts in NER dependent pathway [98]. Only three subunits of TFIIH complex were found in *L. polyedrum* in an analysis of basal transcriptional factors [99]. The absence of putative XPA, RPA3 and GTF2H1 orthologues were also reported in Trypanosomatids [100].

##### Mismatch Repair

**Figure 5:**
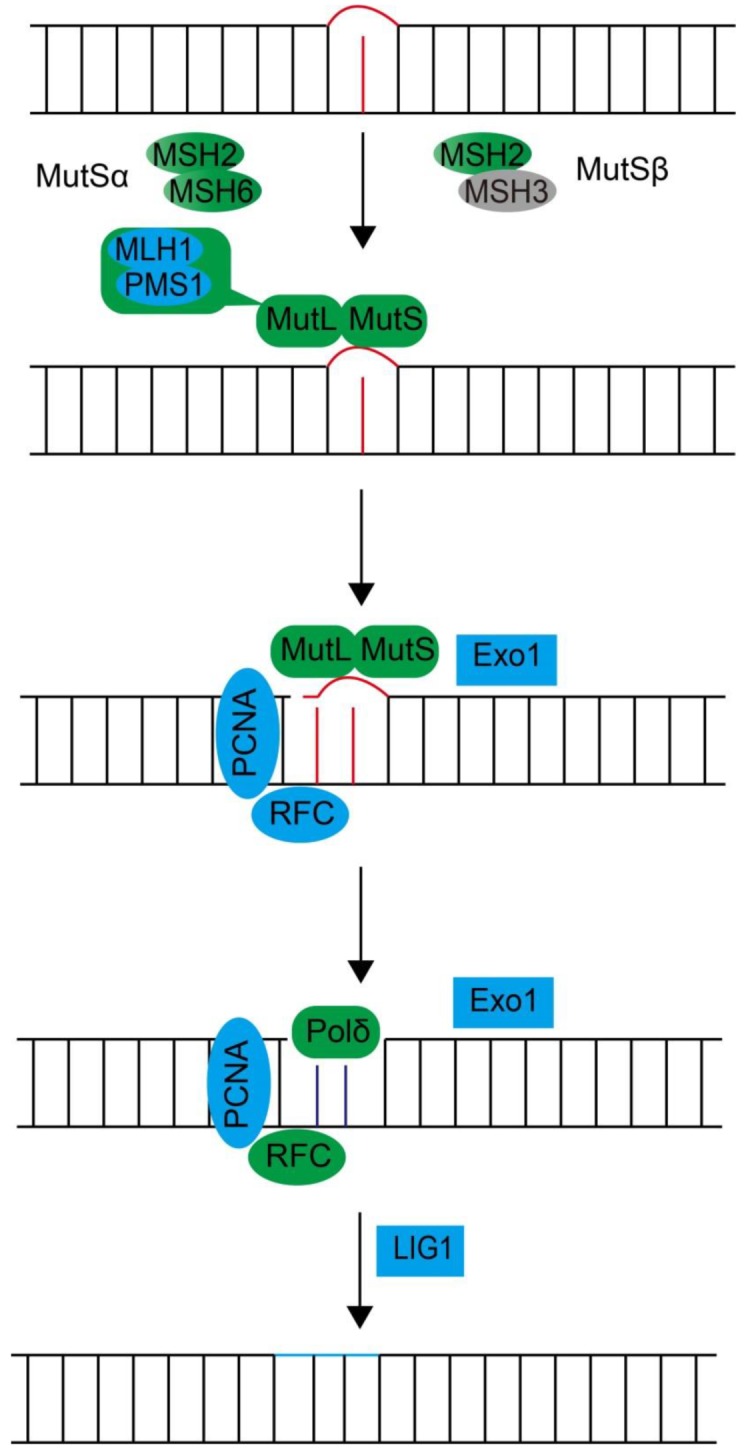
Diagrammatic summary of the dinoflagellate orthologues predicted in the DNA mismatch repair pathway. The rectangles and ellipses filled with grey color indicate the absence of putative dinoflagellate orthologues in the searched transcriptomes. The other colors in rectangles and ellipses indicate the presence of putative dinoflagellate orthologues.

DNA mismatch repair is a DNA repair pathway specializing in repairing mismatched bases during DNA replication, process mismatch-containing heteroduplex during recombination, and correct DNA lesions resulting from either endogenous or exogenous sources [101,102,103,104]. In bacteria, MutS and MutL homodimers are responsible for the initial DNA mismatch recognition, followed by the endonuclease MutH’s nick on the newly synthesized strand. The nicked strand is further excised by the exonuclease and re-synthesized by DNA polymerase [105,106]. Similar eukaryotic mechanisms are also applied for the recognition and repair of DNA mismatches. MutS homologs-membered heterodimers MSH2-MSH6 (MutSα) or MSH2-MSH3 (MutSβ) are used to scan DNA mispair. MutSα has a preference of binding single base mismatches and 1 or 2 base insertions or deletions (indels), while MutSβ is capable of recognizing both base mispairs and small/large indels [101]. The MutL homologs also form heterodimers MLH1-PMS2 (MutLα, also known as MLH1-PMS1 complex in budding yeast cells), which are then recruited to form a ternary complex with MutS homologs to initiate the incision at lesion sites. PCNA protein is thought to activate the endonuclease activities of MutLα and help to distinguish the two DNA strands. Exonuclease 1 (Exo1) subsequently cleaved the nicked mispaired strands and the resulting gap is filled by DNA polymerase Polδ and DNA ligase LIG1. Except MSH3, which is also absent from the apicomplexan *Plasmodium* [107], all the other orthologues are present in dinoflagellates (Figure 5 and Table 5). Detailed mechanism without heterodimer MutSβ awaits further functional investigation.

#### 3.2.4. DNA Double-Strand Breaks Repair

DNA double-strand breaks (DSBs) are one of the most cytotoxic chromosome lesions in which breaks occurs in both parallel strands of duplex DNA. DNA DSBs could be caused by many endogenous and exogenous insults including chromosome replication errors, unintended breakage by nuclear enzymes, reactive species during normal metabolism, ionizing radiation and certain chemical drugs [108]. It has been estimated that DSBs happened spontaneously at a frequency of about one per 10^8^ base pair per cell cycle for many organisms [109]. Additionally, a single unrepaired DNA DSB could lead to cell unviability [110].

Two major distinct pathways are involved in the repair of DSBs: non-homologous end joining (NHEJ) and homologous recombination (HR). NHEJ could occur in the whole cell cycle and particularly during the G1 phase. HR is mainly restricted to in the mid-S and G2 cell-cycle stages, when the nascent sister chromatids are available for recombination repair template.

##### Non-Homologous End Joining

Non-Homologous End Joining (NHEJ) refers to a type of DNA DSB repair that requires little or no sequence homology (≤4 nucleotides) to re-join two DSB ends [111,112]. Binding the DSB ends with the heterodimer Ku protein of Ku70 and Ku80 serves as a loading platform to promote recruitment of other NHEJ proteins [113,114]. Compatible DNA termini such as blunt-ends could be directly repaired with the binding of XRCC4-DNA ligase IV to those termini. For non-compatible ends, the DNA-dependent protein kinase catalytic subunit (DNA-PKcs) is engaged in a complex with DNA nuclease Artemis to process these ends [115,116]. In the complex with DNA-PKcs, Artemis acquires endonuclease activity on the DNA hairpin, and both the 5' and 3' DNA overhangs. Other proteins including aprataxin, APLF and PNK could also execute its function in the modification of these non-compatible ends [117,118,119,120]. Pol X family DNA polymerase, Pol4 in budding yeast [121], PolL and PolM in mammalian cells [122,123], are involved in the nucleotide synthesis during the processing of the ends. In addition, end processing is a repetitive progress, of which modifications could be performed for multiple rounds, leading to a small sequence deletion or insertion [124]. Finally, the ligase IV complex including XRCC4-LIG4 and XLF is responsible for the ligation of the reconstituted compatible ends [125,126,127].

Key players involved in NHEJ, such as Ku70, Ku80, DNA-PK and LIG4 were identified, but orthologues of XLF, XRCC4 and Artemis were not found in dinoflagellates transcriptomes (Table 6). Fission yeast XRCC4 gene is highly divergent from human orthologues and has only been found in recent years [128], whereas XLF orthologues were not found in plants genomes [129]. The lack of full NHEJ pathway was reported in apicomplexan *Plasmodium*, with the absence of Ku70, Ku80, DNA-PK and LIG4 [130].

##### Homologous Recombination

Homologous Recombination (HR) refers to a high-fidelity DSB repair mechanism with the use of a homology sequence as the repair template. In HR, extensive 5′ to 3′ resection of one DNA strand is required at first to produce 3′-OH ended ssDNA tails after DSB formation. DNA termini resection is initiated by the combined action of Mre11-Rad50-NBS1 (MRN) complex and nuclease CtIP, creating a short stretch of ssDNA. Extensive resection is then carried out by additional nucleases and exonucleases including CtIP, DNA2, BLM and EXOI [131,132]. The length of the extensively resected ssDNA tail could range from hundreds to thousands of nucleotides [131]. The RPA protein then binds to the resulting resected ssDNAs to protect it from nucleolytic degradation and formation of secondary structures. In order to proceed to recombination, recombinase Rad51, the central player of HR, is needed to be loaded onto the RPA-coated ssDNA. Mediator proteins, such as Rad52 in yeast and BRCA2 in mammalian cell, are responsible for promoting the Rad51 nucleofilament formation through the displacement of RPA. Other proteins including Rad51 paralogs and Rad54 help to stabilize the Rad51 filaments [133]. Additionally, negative regulators, e.g., DNA helicase Srs2 in yeast, recQ5, BLM and FANCJ in mammalian cells, could suppress Rad51 function via the disassembly of Rad51-ssDNA filaments [134]. Following nucleofilament formation, the recognition of homology sequences and strand invasion ensues, generating a structure called displacement loops (D-loops), primed for DNA repair synthesis from the invading 3'-end ssDNA [135].

In canonical Double-Strand Break Repair (DSBR), the other ssDNA end pairs with the displaced template strand and forms a double Holiday junction (DHJ). The resolution of DHJs could lead to either cross-over or non-crossover recombination products [136]. Alternatively, DHJs formation is inhibited in the synthesis dependent strand annealing (SDSA) mode of HR, in which the D-loops are disrupted after the limited DNA synthesis from the invading 3′-end ssDNA. The displaced 3′-end ssDNA then recombine with the complementary strand of the second 3′-end ssDNA tail, followed by repair DNA synthesis, leading to the formation of non-crossover products. SDSA is the preferred recombination pathway during mitosis [134].

**Figure 6:**
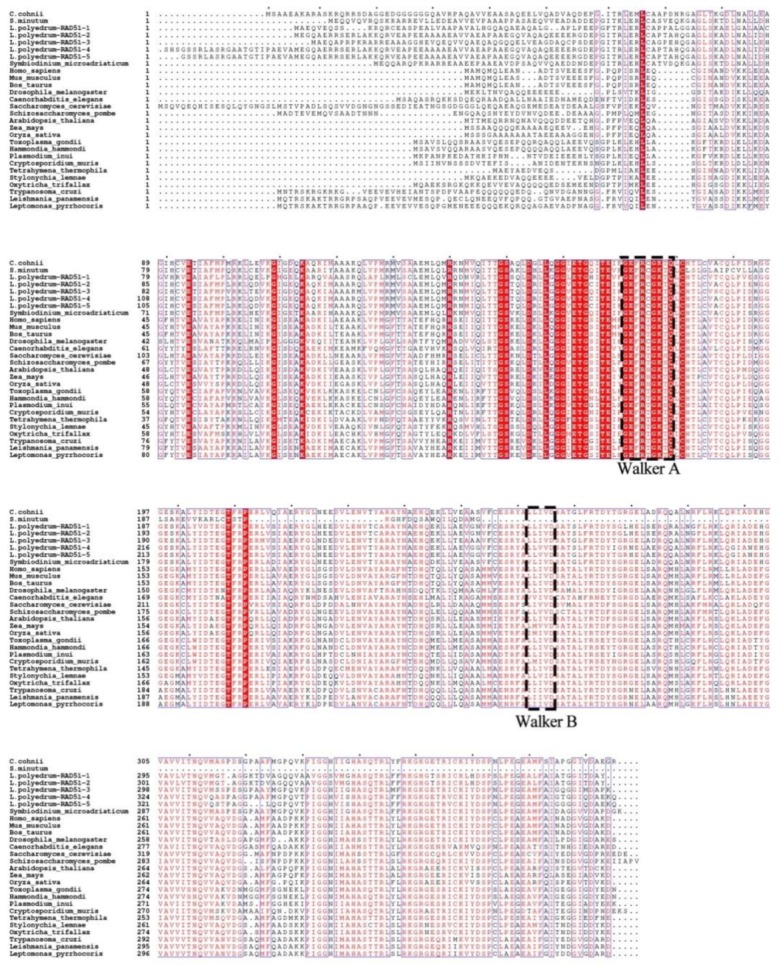
Multi-alignment of dinoflagellate RAD51 proteins.

HR pathway, members of which are also important for meiosis, is uninvestigated at the mechanistic level in dinoflagellates. The essential components of HR including Rad51, MRN complex (MRE11-NBS1-RAD50) and RPA (except RPA3 subunit) could be found in their transcriptomes (Table 7). The mediator protein Rad52 was absent in *Arabidopsis thaliana*, *Drosophila melanogaster*, *Caenorhabditis elegans*, *Plamodium* [130] and *Trypanosomatids* [100], as in the case for dinoflagellates. A putative homolog BRCA2 was found in *Symbiodinum*, which could function as mediator for Rad51. Other putative orthologues such as RMI2, DNA2, SLX4 and EME1 were absent possibly due to the low sequence conservation in dinoflagellates. The recombinase RAD51 responsible for catalyzing the homology search and strand change is the essential protein in the HR pathway [137,138]. Comparative analysis with other eukaryote RAD51 orthologues showed that it contains the canonical Walker A and Walker B motif of the RECA/RAD51 superfamily (Figure 6). A phylogenetic tree constructed with selective eukaryotic orthologues exhibited dinoflagellate RAD51 orthologues forming an individual clade (Figure S2). The prokaryotic RecA orthologues, which are involved in maintaining the integrity of chloroplast and mitochondria genome [139,140], are also present in *S. minutum* and *L. polyedrum* transcriptomes.

#### 3.2.5. Translesion Synthesis

Translesion Synthesis (TLS)—a highly conserved pathway which repairs DNA damages associated with replication—directly mediates duplication of damaged DNA in the template strand that was blocked [141,142,143]. TLS is mediated by the specialized DNA polymerases that could tolerate DNA lesions. These specialized DNA polymerases, including DNA polymerase REV1, Rev3L, PolH, PolI and PolK, have active sites that accommodate impaired or distorted templates [143,144,145]. Execution of TLS involved the switching of canonical polymerases with TLS polymerases, which is thought to be conducted by the interaction with PCNA and Rev1 [146,147]. The low fidelity of TLS polymerase, orthologues of which are found in dinoflagellates transcriptomes (Table 8), makes the repair progress intrinsically error prone [148].

#### 3.2.6. DNA Interstrand Crosslinks Repair

DNA interstrand cross-link (ICL), forming covalent bond between two opposite strands of DNA, can be generated from several sources including bi-functional alkylating agents (such as nitrogen mustard), by-products of lipid peroxidation, abasic sites, and natural psoralens [149]. ICLs prevent complimentary DNA strands separation and thus will impose damages at DNA replication and transcription, making it one of the most toxic DNA damages. In eukaryotes, ICL repair occurs through different mechanisms for non-dividing (G1 phase) and dividing cells (S or G2/M phase) [150,151,152]. However, both mechanisms share similar steps, which include nuclease-mediated detachment from one DNA strand, coupled with TLS polymerase-dependent synthesis across the ICL-containing DNA region, rendering a complete DNA template to finish the repair.

Fanconi anemia is a rare genetic disease associated with the mutation of one of the 19 known *FANC* genes [153]. In cooperation with NER, TLS and HR pathway, the FANC proteins play important roles in signaling and repair of the replication-dependent ICLs [152,154,155]. ICLs recognition is mediated through binding of FANCM to the damaged sites, which function as a landing platform for the recruitment of heptameric FANC core complex (FANCA, FANCB, FANCC, FANCE, FANCF, FANCG and FANCL). The FANC core complex further interacts with many other proteins including other FANC proteins and repair factors to repair the ICLs. It should be mentioned that the full Fanconi anemia pathway genes could to be only found in mammals but not in other organisms. In the yeast *Saccharomyces cerevisiae* and the plant *Arabidopsis thaliana*, a partial Fanconi pathway associated with FANCM was used to repair the ICLs [156,157]. Surprisingly, none of the FANC core complexs, FANCM, and FANCM accessory factors MHF1 and MHF2, were identified in dinoflagellates transcriptomes (Table 9), although we are not certain if their levels at vegetative life cycles may be too rare for mRNA isolation.

The Snm1 family of widely conserved proteins, encoding metallo-b-lactamases (MBL) with nuclease activities, is believed to function in the end procession of the detached ICLs [158,159]. Snm1 deletion in budding yeast showed a hypersensitivity to ICL reagents psoralen and nitrogen mustard [159]. Mammalian cells have three SNM1 orthologues, SNM1A, SNM1B and SNM1C [154], although only SNM1A is thought to be the functional homolog. Both SNM1 and SNM1B were identified in dinoflagellates transcriptomes (Table 9).

## 4. Conclusions and Perspectives

Eukaryotic DNA damage responses (DDR) evolved from—and build on—prokaryotic counterparts, with modifications for nucleosomal accessibility; whether there is a reversal of evolution in dinoflagellates would not only be of interest in evolutionary biology, and potentially bears a biotechnology element for up-scale synthetic biology. We explored the presence of putative orthologues of DDRs genes in three dinoflagellate transcriptomes. We found most of orthologues of DNA damage checkpoint signaling networks, DNA repair pathways including direct reversal of DNA lesion, photoreactivation, BER, NER, mismatch repair, and NHEJ, homologous recombination repair and translesion synthesis, with one exception of Fanconi pathway required for repair of interstrand crosslinks. We speculate that this may be attributed to different forms of DNA damage in anisotropically-aligned supercoiled domains.

Our current knowledge of dinoflagellate DDR is still in its infancy, with a paucity of investigations addressing the specific effect on cell-cycle control, which will directly impact dinoflagellate productivity, and could potentially overlap with the effect of photo-inhibition. The present study represents a starting point for further research in the molecular biology of dinoflagellate DNA damage responses. How DDR coordinates with organelle genome damage processing, in which nuclear genomes seem to have disproportionate representation (especially plastid minicircle) will need to be addressed. In nucleosomal eukaryotes, core histones have major roles in DDRs [160,161]. Their substantially lower expression levels, as well as the lack of nucleosomal nuclease resistance pattern, will have major implications to the molecular mechanism of DNA repair.

## Figures and Tables

**Table 1:** Predicted checkpoint control genes in dinoflagellates.

Genes	Gene ID (E-Value ^#^)			Activity/Remarks
	*C. cohnii*	*S. minutum*	*L. polyedrum*	
Upstream factors				
ATR	Unigene24416_All (2e-022)	symbB.v1.2.040344.t1 (2e-067)	Lp_Unigene54547_All (9e-118)	apical checkpoint serine/threonine kinase
	Unigene40907_All (1e-087)	symbB.v1.2.040393.t1 (1e-018)		
ATRIP	*	*	*	ATR-interacting protein, necessary for checkpoint signaling upon DNA damage
ATM	Unigene1664_All (3e-091)	symbB.v1.2.029032.t1 (1e-087)	Lp_Unigene23700_All (6e-089)	apical checkpoint serine/threonine-protein kinase
Rad17	Unigene30410_All (1e-015)	symbB.v1.2.000094.t1 (7e-008)	Lp_Unigene58859_All (4e-018)	involved in ATR-dependent checkpoint activation
Rad1	CL2244.Contig1_All (2e-024)	-	Lp_Unigene59067_All (6e-006)	subunits of the 9-1-1 complex which binds to DNA lesion after DNA damage
	CL2244.Contig2_All (2e-029)			
Hus1	Unigene57737_All (1e-008)	symbB.v1.2.041619.t1 (3e-014)	Lp_Unigene75437_All (2e-015)	subunits of the 9-1-1 complex which binds to DNA lesion after DNA damage
Rad9	*	*	*	subunits of the 9-1-1 complex which binds to DNA lesion after DNA damage
Mediators				
BRCA1	*	*	*	E3 ubiquitin-protein ligase activity for formation of poly-ubiquitin chains
53BP1	*	*	*	p53-binding protein 1, binds damaged DNA
MDC1	Unigene15380_All (9e-007)	*	Lp_CL4955.Contig1_All (5e-009)	mediator of DNA damage checkpoint, recruitment of repair proteins to DNA damage foci
			Lp_CL4955.Contig2_All ((5e-009))	
Claspin	*	*	*	upstream regulator of Chk1
TopBP1	*	*	Lp_Unigene64350_All (1e-006)	topoisomerase II beta interacting protein, ATR activator
Effectors				
Chk1	*	*	*	serine/threonine-protein kinase
Chk2	Unigene61604_All (3e-056)	symbB.v1.2.014603.t1 (4e-043)	Lp_CL13254.Contig1_All (2e-052)	serine/threonine-protein kinase
	Unigene13287_All (2e-054)	symbB.v1.2.000403.t1 (3e-028)	Lp_Unigene47534_All (3e-041)	
	Unigene68813_All (2e-045)		Lp_CL1544.Contig1_All (6e-049)	
	Unigene52773_All (3e-048)			
	Unigene5075_All (1e-041)			

*, no orthologues could be found; #, E-value obtained from tBLASTn algorithm.

**Table 2:** Predicted dinoflagellate orthologues in direct reversal of DNA damage and photoreactivation.

Genes/Alternate Name or Function Homolog	Gene ID (E-Value ^#^)			Activity/Remarks
	*C. cohnii*	*S. minutum*	*L. polyedrum*	
direct reversal				
MGMT	*	*	Lp_Unigene68135_All (1e-009)	O^6^-methylguanine DNA methyltransferase
			Lp_Unigene71190_All (5e-007)	
ALKB/APH	Unigene40709_All (5e-018)	symbB.v1.2.000566.t1 (1e-006)	Lp_Unigene17309_All (9e-015)	nucleic acid dioxygenase involved in alkylation damage
		symbB.v1.2.012827.t1 (5e-017)		
photoreactivation				
6-4 photolyase	CL2432.Contig2_All (2e-113)	symbB.v1.2.031430.t1 (3e-051)	Lp_Unigene48358_All (9e-128)	involved in repair of (6-4) pyrimidine–pyrimidine induced by UV irradiation
	CL6843.Contig1_All (8e-119)	symbB.v1.2.033063.t1 (5e-114)	Lp_Unigene59149_All (2e-121)	
CPD photolyase	Unigene1971_All (2e-151)	symbB.v1.2.000806.t1 (3e-022)	Lp_Unigene17168_All (3e-015)	involved in repair of cyclobutane-pyrimidine dimer (CPD) induced by UV irradiation
	CL5100.Contig1_All (1e-80)	symbB.v1.2.012126.t1 (6e-027)	Lp_Unigene75562_All (9e-024)	
		symbB.v1.2.023060.t1 (5e-028)	Lp_Unigene21665_All (3e-013)	
		symbB.v1.2.037491.t1 (4e-038)	Lp_Unigene43660_All (3e-012)	
		symbB.v1.2.001247.t1 (7e-041)	Lp_Unigene4570_All (2e-069)	
		symbB.v1.2.030395.t1 (2e-043)	Lp_Unigene41185_All (3e-021)	
		symbB.v1.2.030396.t1 (3e-057)	Lp_Unigene47312_All (2e-074)	
		symbB.v1.2.030397.t1 (6e-022)	Lp_Unigene58611_All (3e-155)	
		symbB.v1.2.031429.t1 (4e-010)	Lp_Unigene63301_All (2e-020)	
		symbB.v1.2.011563.t1 (4e-035)		
cryptochrome DASH	Unigene5536_All (5e-47)	symbB.v1.2.014092.t1 (9e-017)	Lp_Unigene16311_All (3e-108)	removal of cyclobutane-pyrimidine dimers (CPD) in ssDNA
	Unigene41133_All (4e-110)	symbB.v1.2.021362.t1 (8e-068)	Lp_Unigene17251_All (3e-068)	
	Unigene49976_All (5e-69)	symbB.v1.2.025343.t1 (1e-102)	Lp_Unigene20031_All (4e-074)	
		symbB.v1.2.028883.t1 (1e-043)	Lp_Unigene25347_All (9e-078)	
		symbB.v1.2.027391.t1 (7e-046)	Lp_Unigene63655_All (2e-075)	
		symbB.v1.2.021130.t1 (1e-084)	Lp_Unigene67177_All (9e-101)	
		symbB.v1.2.030740.t1 (1e-062)	Lp_CL14399.Contig1_All (2e-097)	
		symbB.v1.2.029072.t1 (1e-035)	Lp_CL14399.Contig2_All (3e-097)	
		symbB.v1.2.015001.t1 (3e-080)	Lp_Unigene7953_All (1e-092)	
		symbB.v1.2.006488.t1 (1e-084)	Lp_Unigene24084_All (4e-065)	
		symbB.v1.2.007315.t1 (2e-026)	Lp_Unigene39631_All (3e-071)	
			Lp_Unigene51086_All (1e-032)	
			Lp_Unigene54913_All (6e-065)	
			Lp_Unigene6860_All (8e-012)	
			Lp_Unigene8874_All (6e-079)	

*, no orthologues could be found; #, E-value obtained from tBLASTn algorithm.

**Table 3:** Predicted dinoflagellate orthologues in base excision repair.

Genes/Alternate Name or Function Homolog	Gene ID (E-Value ^#^)			Activity/Remarks
	*C. cohnii*	*S. minutum*	*L. polyedrum*	
UNG	Unigene44697_All (2e-059)	symbB.v1.2.022126.t1 (9e-069)	Lp_Unigene44751_All (3e-078)	uracil-DNA glycosylases; removes uracil
	Unigene70580_All (6e-071)	symbB.v1.2.022126.t2 (3e-070)	Lp_Unigene47003_All (4e-071)	
		symbB.v1.2.031173.t1 (6e-052)		
SMUG1	*	*	*	single-strand selective mono-functional uracil-DNA glycosylase
MBD4	Unigene51401_All (7e-008)	symbB.v1.2.021865.t1 (2e-014)	Lp_Unigene54655_All (3e-019)	DNA *N*-glycosylase involved in removal of thymine mismatch
TDG	*	*	*	thymine DNA glycosylase; removes thymine mismatch, uracil
OGG1	Unigene85095_All (4e-019)	symbB.v1.2.040865.t1 (5e-011)	Lp_Unigene28278_All (1e-054)	8-oxoG DNA glycosylase
	Unigene82000_All (1e-013)		Lp_Unigene40876_All (4e-054)	
MYH	Unigene67393_All (4e-036)	symbB.v1.2.014787.t1 (4e-067)	Lp_Unigene17300_All (4e-078)	adenine DNA glycosylase; removes A opposite G
NTH1	Unigene62006_All (2e-034)	symbB.v1.2.007780.t1 (4e-050)	Lp_CL9519.Contig1_All (5e-006)	Bi-functional DNA N-glycosylase; removal of oxidized pyrimidines, formamidopyrimidines, 5-formyluracil
	Unigene64896_All (2e-045)	symbB.v1.2.038247.t1 (2e-037)	Lp_Unigene32526_All (1e-061)	
			Lp_Unigene58890_All (6e-053)	
MPG/Mag1	*	symbB.v1.2.024316.t1 (4e-007)	Lp_Unigene52978_All (4e-013)	DNA-3-methyladenine glycosylase
FPG	Unigene42128_All (6e-049)	symbB.v1.2.002485.t1 (2e-071)	Lp_Unigene4975_All (3e-064)	formamidopyrimidine-DNA glycosylase
	CL7240.Contig3_All (3e-048)		Lp_Unigene48155_All (6e-008)	
NEIL1	*	*	*	DNA glycosylases; removal of oxidized pyrimidines, thymine glycol
NEIL2	*	*	*	DNA glycosylases; removal of oxidized pyrimidines
NEIL3	Unigene48672_All (1e-011)	symbB.v1.2.022143.t1 (5e-018)	Lp_Unigene13451_All (5e-014)	DNA glycosylases; removal of oxidized pyrimidines
APE1	Unigene9067_All (1e-033)	symbB.v1.2.023749.t1 (4e-039)	Lp_CL8817.Contig2_All (3e-011)	apurinic/apyrimidinic (AP) endonuclease
	Unigene17453_All (6e-006)	symbB.v1.2.024175.t1 (4e-012)	Lp_CL8837.Contig2_All (1e-036)	
	Unigene34000_All (2e-012)	symbB.v1.2.033468.t1 (1e-035)	Lp_Unigene2242_All (5e-025)	
	Unigene73707_All (2e-011)	symbB.v1.2.033468.t2 (1e-035)	Lp_Unigene33584_All (7e-035)	
	Unigene41862_All (3e-037)			
	Unigene73755_All (1e-032)			
APE2	CL7855.Contig3_All (1e-014)	symbB.v1.2.021745.t1 (1e-009)	Lp_Unigene55979_All (4e-008)	apurinic/apyrimidinic (AP) endonuclease
			Lp_Unigene48320_All (7e-010)	
LIG1	CL2462.Contig1_All (7e-151)	symbB.v1.2.029028.t1 (3e-011)	Lp_CL8189.Contig1_All (9e-022)	DNA ligase required for long-patch BER
	Unigene56781_All (2e-051)	symbB.v1.2.029030.t1 (1e-055)	Lp_CL8189.Contig2_All (6e-043)	
		symbB.v1.2.007861.t1 (7e-021)	Lp_CL13983.Contig1_All (8e-060)	
		symbB.v1.2.007862.t3 (3e-094)	Lp_CL13983.Contig2_All (1e-059)	
		symbB.v1.2.007862.t4 (1e-011)	Lp_Unigene18447_All (6e-020)	
		symbB.v1.2.007862.t5 (5e-081)	Lp_Unigene44117_All (2e-154)	
		symbB.v1.2.007862.t6 (4e-081)		
XRCC1	*	*	*	interacts with PARP, LIG3, and Polβ
LIG3	*	*	*	DNA ligase required for short-patch BER
PKNP	CL5342.Contig1_All (5e-029)		Lp_Unigene21188_All (3e-025)	DNA 3′-phosphatase 5′-kinase required for restoration of 5′-phosphate and 3′-hydroxyl termini
	CL5342.Contig2_All (2e-029)		Lp_Unigene67913_All (4e-012)	
	CL5342.Contig3_All (3e-029)		Lp_Unigene67503_All (8e-027)	
FEN1	Unigene36893_All (6e-102)	symbB.v1.2.012846.t1 (2e-067)	Lp_Unigene74714_All (3e-108)	structure-specific nuclease
		symbB.v1.2.005353.t1 (1e-047)		
		symbB.v1.2.017794.t1 (9e-099)		
PCNA	CL2939.Contig1_All (1e-075)	symbB.v1.2.024689.t1 (1e-069)	Lp_CL16467.Contig1_All (3e-051)	loading platform of PolD1 and PolE1
	CL2939.Contig2_All (3e-075)	symbB.v1.2.005740.t1 (1e-072)	Lp_CL16467.Contig2_All (5e-072)	
	CL2939.Contig3_All (5e-076)	symbB.v1.2.003346.t1 (1e-036)	Lp_CL16467.Contig3_All (2e-039)	
	CL2939.Contig4_All (1e-075)		Lp_CL16467.Contig5_All (4e-051)	
	CL2939.Contig5_All (1e-075)		Lp_CL16467.Contig6_All (3e-056)	
	CL2939.Contig6_All (5e-076)		Lp_CL16467.Contig7_All (6e-057)	
			Lp_CL16467.Contig9_All (9e-058)	
			Lp_Unigene31367_All (2e-075)	
			Lp_Unigene31369_All (3e-072)	
			Lp_CL9592.Contig1_All (1e-008)	
			Lp_CL15065.Contig1_All (1e-011)	
			Lp_CL15065.Contig2_All (1e-010)	
			Lp_CL15065.Contig4_All (5e-010)	
			Lp_Unigene7276_All (2e-033)	
			Lp_Unigene31368_All (7e-029)	
			Lp_Unigene31370_All (1e-027)	
			Lp_Unigene31371_All (2e-022)	
PolB	Unigene65173_All (8e-055)	symbB.v1.2.005179.t1 (8e-051)	Lp_Unigene74664_All (5e-061)	DNA polymerase β/beta
	Unigene70894_All (1e-009)			
POLE1	Unigene29376_All (0.0)	symbB.v1.2.023008.t1 (4e-015)	Lp_CL7119.Contig1_All (0.0)	DNA polymerase ε/epsilon catalytic subunit
	Unigene37482_All (3e-126)	symbB.v1.2.023011.t1 (3e-019)	Lp_CL7119.Contig2_All (0.0)	
		symbB.v1.2.017036.t1 (6e-006)	Lp_Unigene71337_All (5e-029)	
		symbB.v1.2.001887.t1 (7e-131)		
		symbB.v1.2.001887.t2 (2e-130)		
		symbB.v1.2.001887.t3 (3e-048)		
		symbB.v1.2.001889.t1 (1e-042)		
POLD1	Unigene4585_All (3e-050)	symbB.v1.2.029507.t1 (1e-045)	Lp_Unigene28445_All (5e-082)	DNA polymerase δ/delta catalytic subunit
	Unigene44692_All (3e-029)	symbB.v1.2.037870.t1 (2e-089)	Lp_Unigene37178_All (3e-071)	
	Unigene50187_All (8e-055)	symbB.v1.2.036929.t1 (3e-144)	Lp_Unigene55814_All (1e-019)	
	Unigene61374_All (0.0)	symbB.v1.2.036930.t1 (3e-118)	Lp_Unigene70233_All (0.0)	
	Unigene77552_All (2e-148)	symbB.v1.2.033323.t1 (2e-014)	Lp_Unigene71779_All (0.0)	
PARP1	CL1934.Contig3_All (1e-015)	symbB.v1.2.024122.t1 (6e-011)	Lp_CL11756.Contig2_All (2e-006)	poly(ADP-Ribose) polymerase-1; necessary for recruitment of other DNA-repairing enzymes
	Unigene13204_All (1e-027)	symbB.v1.2.018837.t1 (5e-048)	Lp_CL11756.Contig3_All (2e-007)	
	Unigene82709_All (1e-015)		Lp_Unigene29089_All (6e-048)	
			
PARP2	Unigene9060_All (7e-006)	symbB.v1.2.001263.t1 (4e-022)	Lp_CL1803.Contig2_All (5e-028)	poly(ADP-Ribose) polymerase-2; necessary for recruitment of other DNA-repairing enzymes
	Unigene45362_All (7e-017)		Lp_Unigene35603_All (2e-045)	
			Lp_Unigene36239_All (5e-019)	
			Lp_Unigene54635_All (2e-142)	
PARP3	Unigene53685_All (3e-016)	symbB.v1.2.040205.t1 (2e-035)	Lp_Unigene5203_All (6e-032)	poly(ADP-Ribose) polymerase-3; necessary for recruitment of other DNA-repairing enzymes
		symbB.v1.2.034465.t1 (1e-015)		

*, no orthologues could be found; #, E-value obtained from tBLASTn algorithm.

**Table 4:** Predicted dinoflagellate orthologues in nucleotide excision repair.

Genes/Alternate Name or Function Homolog	Gene ID (E-Value ^#^)			Activity/Remarks
	*C. cohnii*	*S. minutum*	*L. polyedrum*	
DDB1	*	symbB.v1.2.027839.t1 (2e-058)	Lp_Unigene56329_All (2e-161)	component of UV-damaged DNA-binding protein complex
DDB2/XPE	*	*	*	component of UV-damaged DNA-binding protein complex
XPC/Rad4	Unigene32549_All (8e-015)	symbB.v1.2.001527.t1 (2e-009)	Lp_Unigene33147_All (3e-017)	binds damaged DNA as XPC complex
Rad23	Unigene21346_All (2e-006)	symbB.v1.2.023245.t1 (2e-022)	Lp_CL13062.Contig2_All (2e-007)	binds damaged DNA as XPC complex
	Unigene68760_All (3e-019)	symbB.v1.2.021833.t1 (6e-017)	Lp_Unigene20125_All (6e-010)	
		symbB.v1.2.010422.t1 (6e-009)	Lp_Unigene72427_All (2e-012)	
			Lp_CL7915.Contig1_All (7e-009)	
			Lp_CL7915.Contig2_All (8e-009)	
			Lp_CL13062.Contig1_All (2e-007)	
			Lp_Unigene36792_All (8e-006)	
			Lp_Unigene50304_All (3e-006)	
CETN2	Unigene29893_All (5e-049)	symbB.v1.2.018116.t1 (5e-043)	Lp_CL4243.Contig1_All (1e-043)	component of the XPC complex
	Unigene38411_All (2e-047)		Lp_CL4243.Contig2_All (7e-043)	
			Lp_CL4243.Contig3_All (2e-043)	
ERCC8/CSA/Rad28	Unigene20816_All (1e-009)	symbB.v1.2.020387.t1 (2e-010)	*	required for transcription-coupled nucleotide excision repair
		symbB.v1.2.020387.t2 (2e-010)		
ERCC-6/Rad26/CSB	Unigene349_All (7e-166)	symbB.v1.2.032523.t1 (5e-084)	Lp_CL6060.Contig1_All (1e-038)	required for transcription-coupled excision repair
	Unigene1702_All (8e-056)	symbB.v1.2.026497.t1 (7e-057)	Lp_CL6060.Contig2_All (2e-036)	
	CL1148.Contig1_All (1e-037)	symbB.v1.2.019527.t1 (1e-161)	Lp_CL12386.Contig2_All (8e-123)	
	Unigene61410_All (2e-041)	symbB.v1.2.019505.t1 (1e-033)	Lp_CL12386.Contig1_All (7e-123)	
		symbB.v1.2.019507.t1 (2e-024)	Lp_CL14187.Contig1_All (6e-157)	
		symbB.v1.2.026498.t1 (3e-021)	Lp_CL14187.Contig2_All (7e-157)	
			Lp_Unigene36232_All (2e-038)	
XPA	*	*	*	binds to DNA damage foci in pre-incision complex
RPA1	Unigene13507_All (3e-041)	symbB.v1.2.000388.t1 (7e-036)	Lp_CL2032.Contig1_All (4e-047)	subunits of heterotrimeric replication protein A complex
	Unigene40788_All (9e-018)	symbB.v1.2.016776.t1 (3e-023)	Lp_CL2032.Contig3_All (2e-048)	
	Unigene69752_All (7e-034)	symbB.v1.2.010569.t1 (9e-043)	Lp_CL2032.Contig4_All (2e-048)	
	Unigene72956_All (1e-043)	symbB.v1.2.015898.t1 (2e-051)	Lp_CL2227.Contig4_All (1e-054)	
			Lp_CL2227.Contig5_All (2e-057)	
			Lp_CL2227.Contig6_All (2e-060)	
			Lp_CL2227.Contig8_All (4e-058)	
			Lp_CL2227.Contig9_All (1e-047)	
			Lp_Unigene5237_All (3e-048)	
			Lp_CL2032.Contig2_All (6e-047)	
			Lp_CL2227.Contig1_All (3e-044)	
			Lp_CL2227.Contig2_All (2e-042)	
			Lp_CL2227.Contig3_All (2e-037)	
			Lp_CL2227.Contig7_All (3e-045)	
			Lp_CL14409.Contig1_All (2e-045)	
			Lp_CL14409.Contig2_All (2e-046)	
			Lp_CL14409.Contig3_All (2e-046)	
			Lp_Unigene27814_All (2e-045)	
RPA2	Unigene14481_All (5e-010)	symbB.v1.2.033139.t1 (3e-007)	Lp_CL6472.Contig2_All (1e-007)	subunits of heterotrimeric replication protein A complex
			Lp_CL6472.Contig5_All (7e-008)	
			Lp_CL6472.Contig6_All (6e-007)	
			Lp_CL9761.Contig1_All (2e-008)	
			Lp_CL9761.Contig2_All (1e-008)	
			Lp_Unigene6245_All (8e-008)	
			Lp_Unigene13916_All (9e-009)	
RPA3	*	*	*	subunits of heterotrimeric replication protein A complex
RFC1	CL4680.Contig1_All (1e-076)	symbB.v1.2.031425.t1 (2e-037)	Lp_CL3410.Contig1_All (2e-050)	required for strand displacement and DNA synthesis
	CL4680.Contig2_All (2e-023)	symbB.v1.2.031427.t1 (9e-013)	Lp_CL3410.Contig3_All (1e-084)	
	CL4680.Contig3_All (2e-076)	symbB.v1.2.000210.t1 (1e-073)	Lp_CL3410.Contig5_All (2e-006)	
	Unigene45646_All (2e-052)	symbB.v1.2.025385.t1 (3e-054)	Lp_CL3410.Contig6_All (3e-016)	
	Unigene77812_All (3e-089)		Lp_Unigene33492_All (2e-008)	
			Lp_Unigene35510_All (6e-040)	
			Lp_Unigene43360_All (3e-049)	
TFIIH complex				catalyzes DNA helix unwinding in pre-incision complex
GTF2H1	*	*	*	core TFIIH subunit p62
GTF2H2	Unigene58152_All (5e-034)	*	Lp_Unigene48571_All (2e-051)	core TFIIH subunit p44
GTF2H3	*	symbB.v1.2.011909.t1 (2e-007)	Lp_Unigene75735_All (6e-014)	core TFIIH subunit p34
GTF2H4	Unigene45546_All (2e-030)	symbB.v1.2.036961.t2 (2e-016)	Lp_Unigene63874_All (5e-022)	core TFIIH subunit p52
GTF2H5	*	*	*	core TFIIH subunit p8
ERCC3/XPB	Unigene17412_All (1e-177)	symbB.v1.2.000409.t1 (1e-150)	Lp_Unigene15679_All (7e-142)	core subunits of TFIIH complex, 3′ to 5′ DNA helicase
ERCC2/XPD/Rad3	Unigene61441_All (0.0)	symbB.v1.2.043770.t1 (3e-017)	Lp_Unigene51894_All (0.0)	core subunits of TFIIH complex, 5′ to 3′ DNA helicase
		symbB.v1.2.003111.t1 (5e-085)		
		symbB.v1.2.003112.t1 (8e-033)		
		symbB.v1.2.003109.t1 (2e-056)		
CDK7	Unigene65253_All (6e-042)	*	Lp_CL3145.Contig1_All (3e-076)	subunit of kinase module of TFIIH complex
	Unigene66528_All (1e-019)		Lp_CL3145.Contig2_All (4e-079)	
			Lp_CL3145.Contig3_All (3e-079)	
MNAT1	Unigene73596_All (2e-014)	symbB.v1.2.023842.t1 (3e-018)	Lp_Unigene66408_All (2e-017)	subunit of kinase module of TFIIH complex
CCNH	*	*	Lp_Unigene28603_All (7e-010)	subunit of kinase module of TFIIH complex
ERCC1	Unigene34115_All (1e-045)	symbB.v1.2.018882.t1 (1e-038)	Lp_Unigene75127_All (5e-053)	5′ incision DNA-binding subunit of TFIIH complex
XPF/ERCC4	Unigene21562_All (2e-035)	symbB.v1.2.008218.t1 (1e-056)	Lp_Unigene48147_All (2e-057)	5′ incision catalytic subunit of TFIIH complex
			Lp_Unigene63025_All (4e-049)	
XPG/ERCC5	Unigene8568_All (2e-031)	symbB.v1.2.006008.t1 (4e-025)	Lp_Unigene10768_All (2e-023)	3′ incision subunits of TFIIH complex
	Unigene57291_All (2e-024)	symbB.v1.2.006011.t1 (2e-026)	Lp_Unigene47869_All (4e-033)	
		symbB.v1.2.006013.t1 (3e-008)		

*, no orthologues could be found; #, E-value obtained from tBLASTn algorithm.

**Table 5:** Predicted dinoflagellate orthologues in DNA mismatch repair.

Genes	Gene ID (E-Value ^#^)			Activity/Remarks
	*C. cohnii*	*S. minutum*	*L. polyedrum*	
MLH1	Unigene48309_All (7e-103)	symbB.v1.2.021543.t1 (3e-111)	Lp_Unigene17816_All (2e-116)	MutL homolog, form heterodimer with PMS1, MLH2 and MLH3
	Unigene56264_All (6e-020)			
MLH3	*	symbB.v1.2.038425.t1 (7e-014)	Lp_Unigene24248_All (7e-036)	MutL homolog, form heterodimer with MLH1
PMS1	CL7093.Contig1_All (1e-042)	symbB.v1.2.037608.t1 (1e-051)	Lp_CL13190.Contig3_All (4e-049)	MutL homolog, form heterodimer with MLH1, part of the DNA mismatch repair (MMR) complex
	CL7093.Contig2_All (9e-044)	symbB.v1.2.037606.t1 (2e-014)	Lp_Unigene124_All (2e-049)	
	CL7093.Contig3_All (3e-041)		Lp_Unigene48112_All (9e-040)	
MSH1	Unigene56453_All (2e-010)	symbB.v1.2.040100.t1 (2e-012)	Lp_Unigene39523_All (1e-006)	MutS homolog, required for repair of mitochondrial DNA
	Unigene41842_All (3e-007)	symbB.v1.2.011567.t1 (4e-019)		
MSH2	Unigene8950_All (3e-133)	symbB.v1.2.002929.t1 (1e-135)	Lp_Unigene75744_All (1e-138)	MutS homolog 2, form heterodimers with MSH3 or MSH6; component of the DNA mismatch repair (MMR) complex
		symbB.v1.2.002929.t2 (2e-061)		
MSH3	*	*	*	MutS homolog 3
MSH6	Unigene36848_All (2e-072)	symbB.v1.2.026874.t1 (1e-056)	Lp_CL3961.Contig1_All (6e-086)	MutS homolog 6
	Unigene62212_All (4e-011)	symbB.v1.2.038785.t1 (5e-012)	Lp_CL3961.Contig2_All (7e-007)	
	Unigene69195_All (3e-023)	symbB.v1.2.039566.t1 (1e-014)	Lp_CL3961.Contig3_All (5e-100)	
			Lp_CL3961.Contig4_All (2e-100)	
			Lp_CL3961.Contig5_All (2e-082)	
			Lp_CL3961.Contig6_All (7e-101)	
MSH4	*	symbB.v1.2.013503.t1 (7e-060)	Lp_Unigene9260_All (8e-065)	MutS homolog 4, specific for meiosis
MSH5	Unigene89588_All (4e-019)	symbB.v1.2.033801.t1 (7e-079)	Lp_Unigene70169_All (1e-039)	MutS homolog 5, specific for meiosis, formation of heterodimer with MSH4
			Lp_Unigene63036_All (2e-030)	
EXO1	Unigene52745_All (3e-035)	symbB.v1.2.019143.t1 (1e-043)	Lp_CL3557.Contig1_All (9e-048)	5′ exonuclease
	CL691.Contig1_All (7e-037)		Lp_Unigene36569_All (2e-037)	
			Lp_Unigene71445_All (9e-056)	
			Lp_Unigene77209_All (1e-007)	

*, no orthologues could be found; #, E-value obtained from tBLASTn algorithm.

**Table 6:** Predicted dinoflagellate orthologues in non-homologous end joining.

Genes/Alternate Name or Function Homolog	Gene ID (E-Value ^#^)			Activity/Remarks
	*C. cohnii*	*S. minutum*	*L. polyedrum*	
Ku70	Unigene61676_All (3e-062)	symbB.v1.2.013334.t1 (2e-048)	Lp_Unigene63133_All (8e-069)	components of Ku heterodimer, which binds to damaged DNA ends
Ku80	Unigene21225_All (8e-012)	symbB.v1.2.017317.t1 (2e-033)	Lp_Unigene60142_All (3e-041)	components of Ku heterodimer, which binds to damaged DNA ends
DNA-PK/PRKDC	Unigene41976_All (4e-076)	symbB.v1.2.025517.t1 (2e-071)	Lp_Unigene62602_All (1e-075)	catalytic subunit of DNA-dependent serine/threonine-protein kinase
	Unigene56338_All (3e-006)			
	Unigene72430_All (3e-006)			
LIG4	Unigene30802_All (5e-018)	symbB.v1.2.033586.t1 (1e-055)	Lp_Unigene8556_All (7e-100)	DNA ligase which forms complex with XRCC4 and responsible for the NHEJ ligation
	Unigene57597_All (3e-023)	symbB.v1.2.033587.t1 (2e-022)		
	Unigene73919_All (3e-029)			
XRCC4	*	*	*	forms complex with LIG4 and responsible for the NHEJ ligation
XLF/Nej1	*	*	*	end-joining factor; serve as bridge for XRCC4-LIG4 complex
Artemis	*	*	*	nuclease for DNA ends processing
APTX	Unigene85214_All (2e-008)	symbB.v1.2.019417.t1 (2e-018)	*	resolve of DNA single-strand interruptions
APLF	*	symbB.v1.2.012028.t1 (7e-006)	*	auxiliary factor for DNA end-joining
PolL	Unigene72723_All (3e-018)	symbB.v1.2.039928.t1 (8e-031)	Lp_Unigene32155_All (3e-036)	DNA polymerase λ/lambda, involved in NHEJ of nuclear DNA
		symbB.v1.2.007119.t1 (2e-037)	Lp_Unigene47083_All (3e-040)	
PolM	*	*	*	DNA polymerase μ/mu
Pol4	*	*	*	DNA polymerase IV

*, no orthologues could be found; #, E-value obtained from tBLASTn algorithm.

**Table 7:** Predicted dinoflagellate orthologues in homologous recombination.

Genes/Alternate Name or Function Homolog	Gene ID (E-Value ^#^)			Activity/Remarks
	*C. cohnii*	*S. minutum*	*L. polyedrum*	
RAD51	Unigene44686_All (1e-133)	symbB.v1.2.024814.t1 (4e-084)	Lp_CL13972.Contig1_All (8e-120)	recombinase, eukaryote RecA homologue
			Lp_CL13972.Contig3_All (6e-123)	
			Lp_CL13972.Contig4_All (6e-126)	
			Lp_CL13972.Contig5_All (2e-124)	
			Lp_Unigene24767_All (2e-110)	
DMC1	Unigene64853_All (9e-113)	symbB.v1.2.000608.t1 (2e-109)	Lp_Unigene20110_All (1e-118)	RAD51 homologue specific for meiosis
		symbB.v1.2.008353.t1 (3e-109)		
RecA	*	symbB.v1.2.015650.t1 (2e-063)	Lp_Unigene51570_All (4e-093)	bacterial recombinase
		symbB.v1.2.028306.t1 (5e-016)		
RAD51B	Unigene1912_All (2e-016)	*	Lp_Unigene71304_All (1e-023)	RAD51 paralog
RAD51C	Unigene41837_All (3e-017)	*	Lp_Unigene66289_All (3e-034)	RAD51 paralog
RAD51D	*	*	*	RAD51 paralog
XRCC2	*	symbB.v1.2.008271.t1 (2e-008)	*	DNA break and cross-link repair
XRCC3	*	*	Lp_Unigene71572_All (2e-010)	DNA break and cross-link repair
RAD50	CL6927.Contig1_All (2e-068)	symbB.v1.2.031416.t1 (3e-051)	Lp_Unigene51516_All (1e-065)	part of the MRN complex
	CL6927.Contig2_All (6e-068)	symbB.v1.2.031416.t2 (3e-051)		
	Unigene31993_All (2e-021)	symbB.v1.2.025527.t1 (8e-044)		
	Unigene68915_All (7e-019)			
NBS1	Unigene77295_All (5e-013)	symbB.v1.2.016565.t1 (3e-014)	Lp_CL4311.Contig2_All (9e-011)	part of the MRN complex
MRE11	Unigene13700_All (8e-117)	symbB.v1.2.022929.t1 (1e-113)	Lp_Unigene63346_All (7e-111)	part of the MRN complex
		symbB.v1.2.022929.t2 (2e-093)		
		symbB.v1.2.022929.t3 (3e-010)		
RAD52	*	*	*	accessory factor for recombination
RAD54L	Unigene77564_All (4e-149)	symbB.v1.2.021721.t2 (3e-127)	Lp_CL12947.Contig4_All (4e-148)	involved in recombination
		symbB.v1.2.012979.t1 (9e-088)	Lp_Unigene46377_All (6e-147)	
		symbB.v1.2.021720.t1 (4e-077)	Lp_CL12947.Contig2_All (2e-148)	
		symbB.v1.2.021721.t1 (4e-066)	Lp_CL12947.Contig3_All (2e-148)	
		symbB.v1.2.021721.t3 (4e-066)		
RAD54B	*	symbB.v1.2.012978.t1 (6e-024)	Lp_Unigene31872_All (5e-096)	accessory factor for recombination
			Lp_Unigene981_All (4e-037)	
BRCA2	*	symbB.v1.2.003783.t1 (1e-008)	*	involved in recombination
DSS1/SHFM1	*	*	*	BRCA2 accessary factor
CtIP	*	symbB.v1.2.018901.t1 (3e-006)	*	involved in DNA end resection
BLM/Sgs1	*	symbB.v1.2.009075.t3 (2e-037)	Lp_Unigene24386_All (1e-018)	3′-5′ DNA helicase
		symbB.v1.2.009075.t4 (2e-037)		
		symbB.v1.2.009075.t2 (2e-037)		
		symbB.v1.2.009074.t1 (3e-041)		
		symbB.v1.2.008709.t1 (7e-022)		
Top3α	Unigene32503_All (2e-163)	symbB.v1.2.026949.t1 (6e-160)	Lp_Unigene1654_All (3e-166)	DNA topoisomerase 3-alpha
		symbB.v1.2.035328.t1 (2e-021)	Lp_Unigene44680_All (6e-022)	
		symbB.v1.2.037631.t1 (2e-006)	Lp_CL12602.Contig1_All (3e-011)	
			Lp_Unigene21536_All (4e-012)	
			Lp_Unigene29037_All (1e-011)	
			Lp_Unigene53944_All (3e-006)	
			Lp_Unigene62514_All (3e-012)	
RMI1	Unigene5006_All (6e-010)	symbB.v1.2.037665.t1 (1e-006)	Lp_Unigene13746_All (1e-010)	key component of the RMI complex
RMI2	*	*	*	key component of the RMI complex
DNA2	*	*	*	ATP-dependent helicase/nuclease
PARI/Srs2	Unigene16314_All (1e-009)	*	*	inhibit inappropriate homologous recombination
MUS81	Unigene1977_All (6e-016)	symbB.v1.2.018987.t1 (1e-017)	Lp_Unigene50884_All (1e-013)	subunit of structure-specific endonuclease
EME1/MMS4	*	*	*	interaction with Mus81
SLX1	Unigene40330_All (4e-021)	symbB.v1.2.003012.t1 (1e-020)	Lp_Unigene20489_All (2e-020)	subunit of SLX1-SLX4 structure-specific nuclease
		symbB.v1.2.009695.t1 (2e-012)		
SLX4	*	*	*	subunit of SLX1-SLX4 structure-specific nuclease
GEN1/YEN1	Unigene8182_All (2e-008)	*	Lp_Unigene2100_All (7e-019)	nuclease seperating Holliday junctions
			Lp_Unigene17855_All (2e-012)	
SPO11	Unigene18126_All (3e-012)	symbB.v1.2.038121.t1 (7e-025)	Lp_Unigene20651_All (2e-024)	meiosis specific endonuclease
	Unigene24373_All (1e-012)	symbB.v1.2.012520.t1 (8e-031)	Lp_Unigene9316_All (2e-038)	

*, no orthologues could be found; #, E-value obtained from tBLASTn algorithm.

**Table 8:** Dinoflagellate orthologues predicted in translesion synthesis.

Genes/Alternate Name or Function Homolog	Gene ID (E-Value ^#^)			Activity/Remarks
	*C. cohnii*	*S. minutum*	*L. polyedrum*	
Rev3L/PolZ	Unigene7171_All (1e-017)	symbB.v1.2.025103.t1 (1e-067)	Lp_Unigene29950_All (8e-022)	DNA polymerase ζ/zeta catalytic subunit
	Unigene32515_All (9e-131)	symbB.v1.2.025103.t2 (3e-067)	Lp_Unigene60556_All (1e-025)	
	Unigene56596_All (3e-083)	symbB.v1.2.025103.t3 (2e-067)	Lp_Unigene61611_All (1e-030)	
		symbB.v1.2.028081.t1 (7e-091)	Lp_Unigene76685_All (4e-026)	
		symbB.v1.2.028084.t1 (1e-024)	Lp_Unigene76716_All (1e-013)	
			Lp_Unigene81304_All (2e-008)	
REV1	Unigene56396_All (3e-046)	symbB.v1.2.017539.t1 (2e-014)	Lp_Unigene31865_All (3e-008)	non-classical DNA polymerase, dCMP transferase
		symbB.v1.2.017542.t1 (1e-017)	Lp_Unigene55084_All (5e-053)	
			Lp_Unigene62480_All (6e-044)	
PolH/Rad30	Unigene678_All (9e-062)	symbB.v1.2.015189.t1 (3e-054)	Lp_Unigene8962_All (3e-049)	DNA polymerase η/eta involved in the DNA repair by translesion synthesis
	Unigene54870_All (1e-008)	symbB.v1.2.015189.t2 (9e-051)		
		symbB.v1.2.017537.t1 (3e-027)		
PolI/Rad30B	Unigene46925_All (8e-036)	symbB.v1.2.027247.t1 (6e-058)	Lp_Unigene39489_All (1e-056)	error-prone DNA polymerase ι/iota involved in bypass of DNA lesions
PolK/DINB1	Unigene49999_All (1e-044)	symbB.v1.2.024275.t1 (1e-016)	Lp_Unigene16086_All (8e-040)	error-prone DNA polymerase κ/kappa involved in bypass of DNA lesions

#, E-value obtained from tBLASTn algorithm.

**Table 9:** Predicted dinoflagellate orthologues predicted in interstrand crosslinks repair.

Genes	Gene ID (E-Value ^#^)			Activity/Remarks
	*C. cohnii*	*S. minutum*	*L. polyedrum*	
FANCA	*	*	*	core complex member required for interstrand DNA cross-link repair
FANCB	*	*	*	*ibid*
FANCC	*	*	*	*ibid*
FANCE	*	*	*	*ibid*
FANCF	*	*	*	*ibid*
FANCG	*	*	*	*ibid*
FANCL	*	*	*	*ibid*
FANCM	*	*	*	lesion recognition and recruitment of the core complex
MHF1	*	*	*	involved in the damage-dependent DNA binding of FANCM
MHF2	*	*	*	involved in the damage-dependent DNA binding of FANCM
SNM1	Unigene68129_All (9e-006)	symbB.v1.2.005478.t1 (5e-046)	Lp_Unigene56381_All (2e-063)	required for interstrand DNA cross-link repair
	Unigene48769_All (6e-023)			
SNM1B	*	symbB.v1.2.023872.t2 (1e-024)	Lp_Unigene44216_All (4e-036)	related to SNM1

*, no orthologues could be found; #, E-value obtained from tBLASTn algorithm.

## Supplementary Materials

The following are available online at https://www.mdpi.com/2076-2607/7/7/191/s1. Table S1. Sequences of extracted putative DDR orthologues from *C. cohnii* and *L. polyedrum*.
