# Supplementary material for: DNA Damage Response Pathways in Dinoflagellates
Source: Microorganisms. 2019 Jul 5;7(7):191. doi: 10.3390/microorganisms7070191 (PMC6680887; doi:10.3390/microorganisms7070191)
Supplement: Supplementary file 1 [file microorganisms-07-00191-s001.zip › microorganisms-506447 supplementary figures.pdf]

(A) ATM protein

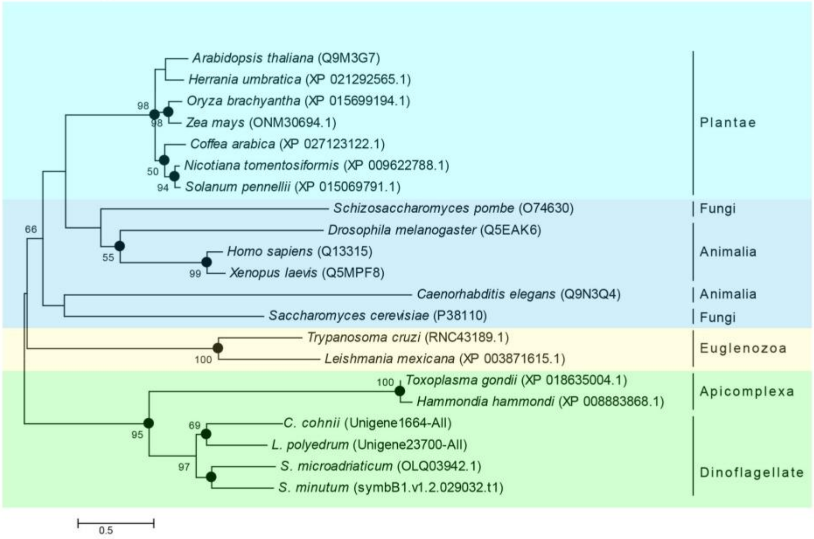

(B) ATR ptotein

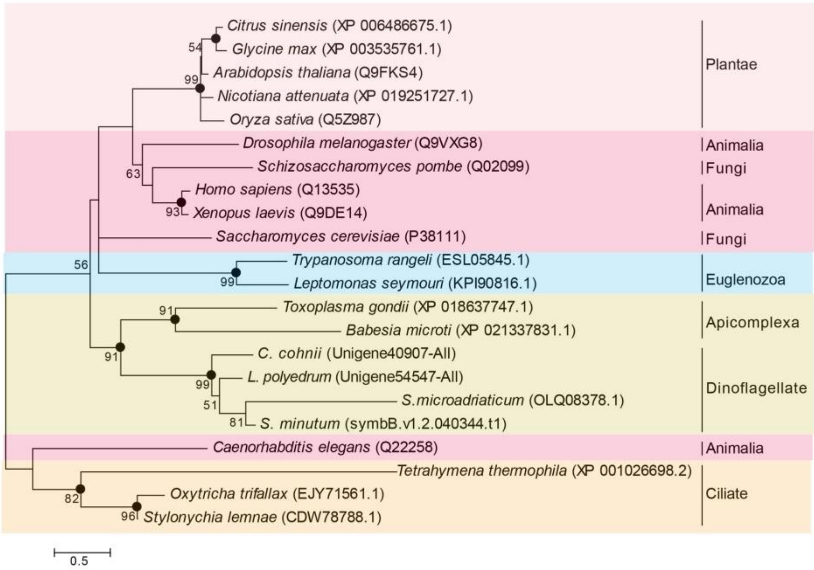

Figure S1. Phylogenetic analysis of dinoflagellate central checkpoint kinases ATM and ATR. (**A**) Maximum-likelihood phylogenetic tree based on the analysis of dinoflagellate and representative eukaryotes ATM sequences; (**B**) Maximum-likelihood phylogenetic tree based on the analysis of dinoflagellate and representative eukaryote ATR sequences. Bootstrap values (>50%, with 1000 replications of resampling analysis) are shown at branch points. Filled circles indicate that the nodes were also recovered by trees generated using the neighbour-joining method. GenBank accession numbers and dinoflagellate protein ID numbers are included in the bracket.

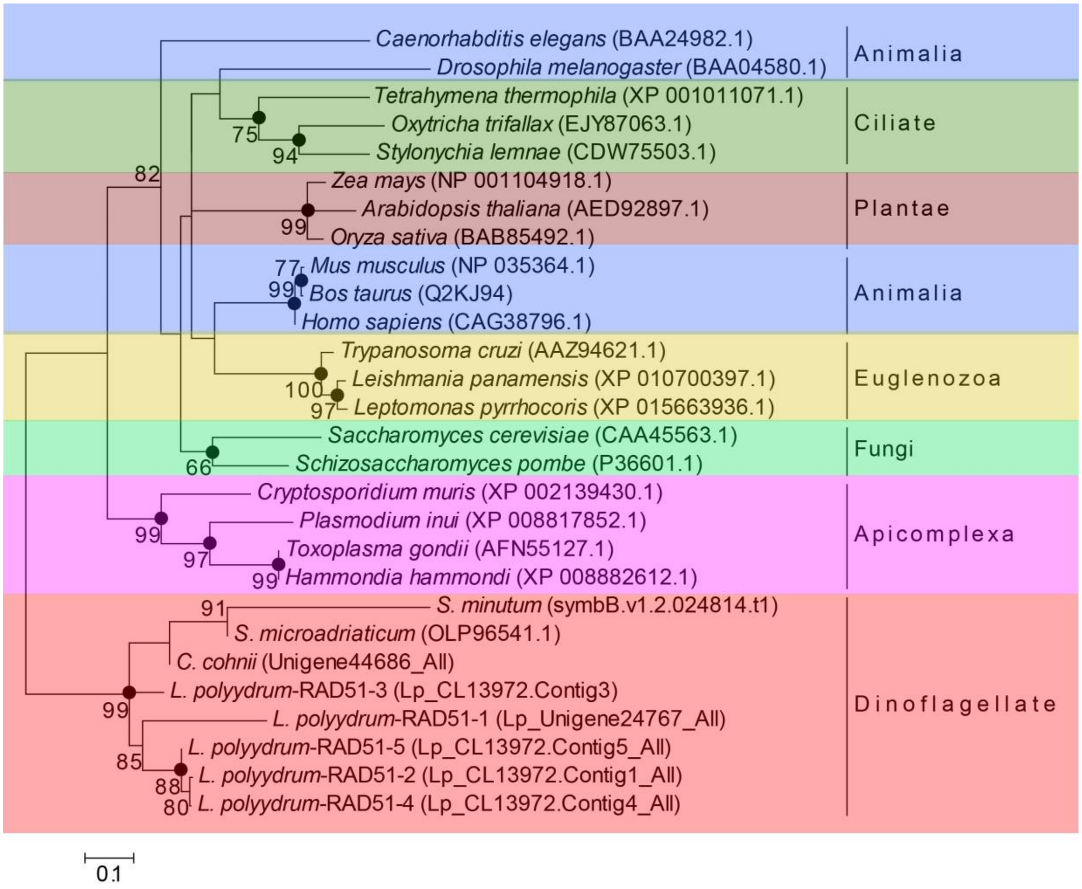

Figure S2. Phylogenetic analysis of dinoflagellates Rad51 proteins. Maximum-likelihood phylogenetic tree based on the analysis of dinoflagellate and representative eukaryotes Rad51 protein sequences. Bootstrap values (>50%, with 1000 replications of resampling analysis) are shown at branch points. Filled circles mean that the nodes were also recovered by trees generated using the neighbour-joining method. GenBank accession numbers and dinoflagellate protein ID numbers are included in the bracket.
